# An interpretable ensemble learning framework for COPD classification using population-based clinical and laboratory data from NHANES

**DOI:** 10.18332/tid/225230

**Published:** 2026-07-23

**Authors:** Haiting Tong, Jintao Huang, Bin Zhang, Xiangxiang Fu

**Affiliations:** 1 Yuyao Maternity and Child Health Care Hospital, Yuyao Second People's Hospital, Ningbo, China

**Keywords:** chronic obstructive pulmonary disease, machine learning, explainable artificial intelligence, population-based screening

## Abstract

**INTRODUCTION:**

Chronic obstructive pulmonary disease (COPD) represents the third leading cause of death globally, affecting 213.39 million people, yet approximately 70% of cases remain undiagnosed due to insufficient spirometry utilization in primary care settings.

**METHODS:**

We developed machine learning-based COPD classification models using nationally representative data from 8061 participants aged ≥40 years in the National Health and Nutrition Examination Survey 2007–2012. Least Absolute Shrinkage and Selection Operator (LASSO) regression with repeated ten-fold cross-validation systematically selected sixteen features from 33 candidate variables. We compared twelve machine learning algorithms. SHapley Additive exPlanations (SHAP) analysis provided model interpretability.

**RESULTS:**

CatBoost demonstrated optimal performance on the validation set with an area under the receiver operating characteristic curve (AUC) of 0.788, a sensitivity of 0.706, and a specificity of 0.728. SHAP analysis identified age and smoking duration as dominant risk factors, revealing non-linear relationships, including body mass index's U-shaped association with COPD risk and synergistic age-smoking interactions. Decision Curve Analysis confirmed net clinical benefit across risk thresholds of 0.1 to 0.6.

**CONCLUSIONS:**

This study establishes a machine learning framework combining systematic feature selection, comprehensive algorithm evaluation, and explainable artificial intelligence for COPD risk classification using readily available clinical data. The model achieved a clinically appropriate sensitivity-specificity balance through threshold optimization, demonstrating potential for risk-stratified spirometry referral in primary care settings. External validation in independent populations represents the essential next step before clinical implementation.

## INTRODUCTION

Chronic obstructive pulmonary disease (COPD) represents a critical global health challenge. As the third leading cause of death worldwide, COPD affected 213.39 million people in 2021, with projections approaching 600 million cases by 2050^1^. The gold standard for diagnosis, post-bronchodilator spirometry, is substantially underutilized in primary care settings due to limited access, lack of provider familiarity with guidelines, and insufficient testing proficiency^2^. This diagnostic gap necessitates accessible, non-invasive risk classification tools using readily available clinical indicators to identify high-risk individuals for targeted spirometry referral. Advanced machine learning approaches applied to population-based data, offer a promising strategy to bridge this gap.

Traditional regression models for COPD risk classification achieve only moderate discrimination, with pooled C-statistics of 0.62–0.77^3^. These approaches struggle with high-dimensional data, complex variable interactions, and non-linear relationships. While machine learning algorithms, particularly ensemble methods, have shown superior performance with AUC values ranging from 0.72 to 0.86, existing models have important limitations. Most utilize small, single-center datasets from specialized respiratory clinics^4^, limiting generalizability to primary care populations. Few studies leverage nationally representative data capturing the full disease severity spectrum. Variable selection is often empirical rather than systematic, risking overfitting. Comprehensive assessment of inflammatory and nutritional biomarkers remains limited despite their recognized prognostic value in COPD. These gaps necessitate more rigorous, population-based approaches.

This study addresses these limitations through a systematic methodological framework integrating LASSO-based feature selection, comprehensive machine learning algorithm comparison, and explainable artificial intelligence analysis. We analyzed 8061 participants aged ≥40 years from the National Health and Nutrition Examination Survey (NHANES) 2007–2012 with post-bronchodilator spirometry meeting American Thoracic Society (ATS)/European Respiratory Society (ERS) quality standards (grades A or B). We hypothesized that systematic feature selection combined with ensemble learning would achieve superior discrimination while maintaining interpretability for risk-stratified spirometry referral in primary care settings.

## METHODS

### Study design

This secondary dataset diagnostic-classification modeling study with internal validation utilized cross-sectional data from the NHANES spanning three consecutive survey cycles from 2007 to 2012. This specific timeframe was selected because only these three cycles contained complete pulmonary function test data, including measurements of forced expiratory volume in 1 second (FEV_1_) and forced vital capacity (FVC). NHANES employs a complex multistage probability sampling design to ensure national representativeness of the non-institutionalized civilian population of the United States. The NHANES study protocol received approval from the Research Ethics Review Board of the National Center for Health Statistics (NCHS), and all participants provided written informed consent.

Inclusion criteria required participants to have completed pulmonary function testing that met ATS/ ERS standards, with test quality grades of A or B. Exclusion criteria encompassed missing pulmonary function data or quality grades of C or D, aged <40 years, and pregnant women. The latter group was excluded because physiological pulmonary function changes during pregnancy may compromise the accuracy of FEV_1_/FVC measurements.

From the initial sample of 30442 individuals across the three NHANES cycles from 2007 to 2012, we first excluded 11366 participants with missing pulmonary function data, followed by the exclusion of 11015 participants who were either younger than 40 years or pregnant women. This yielded a final analytical sample of 8061 eligible participants.

### Analytical strategy

This study employed three methodological approaches for COPD classification. First, LASSO regression with repeated 10-fold cross-validation was used to systematically select optimal features from 33 candidate variables spanning demographics, anthropometrics, lifestyle factors, comorbidities, and laboratory biomarkers. LASSO’s L1 regularization prevents overfitting while enhancing model parsimony. Second, 12 machine learning algorithms were compared, including traditional methods (logistic regression, linear discriminant analysis) and ensemble approaches (Random Forest, XGBoost, CatBoost, LightGBM), to identify the optimal classification framework. Third, SHAP analysis was applied to quantify individual feature contributions, revealing non-linear relationships that traditional methods cannot detect. Clinical utility was evaluated through Decision Curve Analysis.

### Outcome variable definition

This study employed Global Initiative for Chronic Obstructive Lung Disease (GOLD) guideline-recommended spirometric criteria to define COPD^5,6^, whereby a post-bronchodilator FEV_1_/FVC ratio of <0.70 was classified as COPD and a ratio of ≥0.70 as non-COPD. Spirometry was performed using calibrated Ohio 822/827 dry-rolling seal spirometers following ATS standardized protocols. Eligible participants received albuterol and underwent post-bronchodilator spirometry after a minimum 10-minute waiting period. Participants performed repeated maximal forced expiratory maneuvers until achieving reproducible results, with a maximum of eight attempts permitted. The highest FEV_1_ and FVC values from acceptable maneuvers were selected to calculate the FEV_1_/FVC ratio. To ensure data quality, this study exclusively included spirometry measurements with quality grades of A or B, where Grade A exceeded ATS standards and Grade B met ATS standards for reproducibility.

### Feature variable selection

Based on existing literature and clinical expertise, this study systematically extracted 36 candidate feature variables associated with COPD from the NHANES database^7,8^. These variables encompassed multiple dimensions, including demographic characteristics (age, sex, race/ethnicity, education level, and poverty-income ratio), anthropometric measurements (body mass index and waist circumference), lifestyle factors (smoking status, smoking years, and physical activity level), comorbidities (asthma, cardiovascular disease, hypertension, diabetes, and chronic kidney disease [CKD]), and laboratory indicators.

Smoking status was categorized as never smoker (<100 cigarettes in lifetime), current smoker (≥100 cigarettes in lifetime and currently smoking), or former smoker (≥100 cigarettes in lifetime but not currently smoking). Laboratory indicators were further subdivided into hematological parameters (hemoglobin, hematocrit, white blood cell count, neutrophil percentage, lymphocyte percentage, and eosinophil percentage), inflammatory markers (neutrophil-to-lymphocyte ratio [NLR], platelet-to-lymphocyte ratio [PLR], and systemic immune-inflammation index [SII]), nutritional indicators (serum albumin, vitamin D, advanced lung cancer inflammation index [ALI], geriatric nutritional risk index [GNRI], and prognostic nutritional index [PNI]), lipid profile (total cholesterol, high-density lipoprotein cholesterol [HDL-C], and triglycerides), hepatorenal function indicators (alanine aminotransferase [ALT] and estimated glomerular filtration rate [eGFR]), and glucose metabolism indicators (fasting glucose and glycated hemoglobin). All laboratory examinations were performed at the NHANES Mobile Examination Center by certified technicians following standardized operating procedures, with rigorous quality control measures implemented throughout.

### Data preprocessing

This study employed a systematic approach to handle missing data. After assessing missingness, three variables (triglycerides, fasting glucose, glycated hemoglobin) exceeded 10% missing rate and were excluded.

The dataset was randomly partitioned into training and validation sets at a 7:3 ratio. The training set comprised 70% of the total sample and was utilized for model training and hyperparameter optimization. The validation set comprised the remaining 30% of the sample and served as an independent validation set for final model performance evaluation. The validation set data remained completely unseen during the model training and hyperparameter optimization processes and was employed solely for independent performance verification after all model training was completed, thereby ensuring the objectivity and unbiasedness of the evaluation results.

For the remaining variables with minimal missing values, different imputation methods were applied according to variable type, with all imputation and standardization parameters derived exclusively from the training set and subsequently applied to the validation set. For continuous variables, distribution characteristics were first assessed using the Shapiro-Wilk test. Variables following a normal distribution were imputed using mean imputation, while those exhibiting skewed distributions were imputed using median imputation to enhance robustness. For categorical variables, the K-Nearest Neighbors (KNN) imputation method was employed with K set to 5, selecting the most similar neighboring samples based on Euclidean distance to infer missing values. These conservative single imputation methods were chosen because: 1) missing rates were minimal across all retained variables; and 2) neither mean/median nor KNN imputation incorporates outcome information, thereby minimizing the risk of information leakage. Additionally, to eliminate the influence of different measurement scales and numerical ranges across variables, all continuous variables underwent Z-score standardization, while categorical variables retained their original coding format.

### LASSO-based variable selection

To avoid overfitting and multicollinearity issues arising from high-dimensional data, this study employed the Least Absolute Shrinkage and Selection Operator (LASSO) logistic regression to perform feature selection on the 33 candidate variables. LASSO introduces an L1 regularization penalty term into the loss function, which automatically shrinks the regression coefficients of unimportant variables to zero, thereby achieving feature dimensionality reduction and model simplification. This study implemented LASSO regression using the *glmnet* package in R software, with the optimal regularization parameter λ determined through repeated 10-fold cross-validation (five repetitions). According to the one-standard-error rule, lambda.1se was selected as the final regularization parameter, which corresponds to the largest λ value within one standard error of lambda.min (the λ value that minimizes cross-validation error). This strategy yielded a more parsimonious and robust model while sacrificing minimal classification accuracy. To visualize the variable selection process, LASSO coefficient path plots and cross-validation error curves were generated. Based on the lambda.1se criterion, 16 variables with non-zero regression coefficients were successfully selected from the 33 candidate variables as the final feature set for subsequent machine learning model construction.

### Machine learning model construction

Based on the 16 features variables selected by LASSO from the training set, this study constructed 12 different types of machine learning models to comprehensively compare their performance in COPD classification tasks. These models included logistic regression (LR) as the traditional baseline model, linear discriminant analysis (LDA) for linear classification, support vector machine with radial basis function kernel (SVM-RBF) to handle nonlinear relationships, K-Nearest Neighbors (KNN) as an instance-based nonparametric method, and neural network (NN) to learn complex feature representations through multilayer perceptrons. Additionally, this study emphasized the evaluation of multiple ensemble learning algorithms, including Random Forest (RF), Gradient Boosting Machine (GBM), Extreme Gradient Boosting (XGBoost), Light Gradient Boosting Machine (LightGBM), Categorical Boosting (CatBoost), Adaptive Boosting (AdaBoost), and Partial Least Squares Discriminant Analysis (PLS-DA).

All hyperparameter tuning was performed exclusively on the training set using grid search with 5-fold cross-validation; the validation set remained completely unseen during model development. For models implemented within the caret framework, hyperparameters were optimized using classification accuracy as the selection criterion, with algorithm-specific tuning grids defined for each model (e.g. *mtry* for Random Forest, interaction depth and number of trees for GBM, K for KNN). CatBoost and LightGBM were tuned using their native implementations with custom grid search over key hyperparameters (tree depth, learning rate, and regularization coefficients), using 5-fold cross-validation with AUC as the optimization metric and early stopping (patience of 50 rounds, maximum 500 iterations) to prevent overfitting. Final models were retrained on the full training set with optimal hyperparameters and then evaluated on the held-out validation set.

NHANES sampling weights were incorporated in all descriptive analyses (Tables 1 and 2) to ensure nationally representative population estimates, but were not applied during machine learning model training. The primary modeling objective was to optimize individual-level classification accuracy rather than to estimate population-level parameters, and incorporating complex survey weights into iterative machine learning algorithms lacks established theoretical frameworks and standardized implementations.

**Table 1:** Baseline characteristics of participants included in the NHANES 2007–2012 analytic sample, stratified by training and validation sets (N=8061)

*Characteristics*	*Overall* *(N=8061)*	*Training set* *(N=5643)*	*Validation set* *(N=2418)*	*p*
**COPD**	1520 (19.89)	1064 (20.10)	456 (19.40)	0.554
**Age** (years)	54.00 (47.00–62.00)	54.00 (47.00–63.00)	54.00 (47.00–62.00)	0.496
**Gender**				0.482
Male	4017 (48.44)	2815 (48.76)	1202 (47.69)	
Female	4044 (51.56)	2828 (51.24)	1216 (52.31)	
**Race/Ethnicity**				0.873
Mexican American	1172 (5.96)	828 (6.07)	344 (5.69)	
Other Hispanic	863 (4.36)	591 (4.26)	272 (4.60)	
Non-Hispanic White	3619 (73.58)	2516 (73.51)	1103 (73.74)	
Non-Hispanic Black	1800 (10.24)	1271 (10.32)	529 (10.05)	
Other race	607 (5.86)	437 (5.84)	170 (5.91)	
**BMI** (kg/m^2^)	28.28 (24.88–32.50)	28.38 (25.00–32.65)	28.04 (24.60–32.21)	0.124
**PNI**	52.75 (50.00–55.36)	52.75 (50.03–55.35)	52.69 (49.93–55.36)	0.665
**Hemoglobin**	14.30 (13.50–15.20)	14.30 (13.50–15.20)	14.20 (13.40–15.20)	0.490
**Vitamin D**	67.80 (54.60–85.70)	67.20 (54.30–84.90)	68.80 (55.30–86.60)	0.095
**Lymphocytes** (%)	29.60 (24.60–34.20)	29.60 (24.50–34.30)	29.60 (24.70–34.00)	0.999
**Eosinophils** (%)	2.50 (1.70–3.50)	2.50 (1.70–3.50)	2.50 (1.60–3.50)	0.203
**ALT**	23.00 (18.00–29.00)	23.00 (18.00–29.00)	22.00 (18.00–28.00)	0.220
**Smoking status**				0.322
Never	4092 (51.66)	2856 (51.04)	1236 (53.13)	
Former	2336 (29.34)	1640 (29.45)	696 (29.09)	
Current	1633 (19.00)	1147 (19.51)	486 (17.77)	
**Smoking years**	0.00 (0.00–24.00)	0.00 (0.00–24.00)	0.00 (0.00–23.00)	0.194
**Physical activity**				0.369
Low	4909 (57.36)	3431 (57.00)	1478 (58.20)	
Moderate	1759 (23.56)	1211 (23.42)	548 (23.89)	
High	1393 (19.08)	1001 (19.57)	392 (17.91)	
**Asthma**	979 (12.59)	687 (12.55)	292 (12.69)	0.905
**Diabetes**	1248 (11.24)	879 (11.39)	369 (10.89)	0.631
**CKD**	630 (5.71)	452 (5.97)	178 (5.10)	0.199

Data are presented as n (unweighted count) and % (weighted percentage) for categorical variables, and median (interquartile range) for continuous variables. P-values were calculated using the Rao-Scott chi-squared test for categorical variables and the survey-weighted Wilcoxon rank-sum test for continuous variables, accounting for the NHANES complex survey design. COPD, chronic obstructive pulmonary disease. BMI: body mass index. PNI: prognostic nutritional index. ALT: alanine aminotransferase (U/L). CKD: chronic kidney disease. Statistical significance was defined as p<0.05.

**Table 2:** Comparison of baseline characteristics between COPD and non-COPD groups in the NHANES 2007–2012 analytic sample (N=8061)

*Characteristics*	*Overall* *(N=8061)*	*Non-COPD* *(N=6541)*	*COPD* *(N=1520)*	*p*
**Age** (years)	54.00 (47.00–62.00)	52.00 (46.00–61.00)	59.00 (52.00–67.00)	<0.001
**Gender**				<0.001
Male	4017 (48.44)	3051 (45.61)	966 (59.85)	
Female	4044 (51.56)	3490 (54.39)	554 (40.15)	
**Race/Ethnicity**				<0.001
Mexican American	1172 (5.96)	1063 (6.91)	109 (2.13)	
Other Hispanic	863 (4.36)	761 (4.94)	102 (2.02)	
Non-Hispanic White	3619 (73.58)	2697 (70.99)	922 (84.00)	
Non-Hispanic Black	1800 (10.24)	1492 (10.90)	308 (7.62)	
Other race	607 (5.86)	528 (6.26)	79 (4.23)	
**BMI** (kg/m^2^)	28.28 (24.88–32.50)	28.69 (25.19–32.93)	26.90 (23.90–30.60)	<0.001
**PNI**	52.75 (50.00–55.36)	52.75 (50.10–55.41)	52.49 (49.67–55.09)	0.043
**Hemoglobin**	14.30 (13.50–15.20)	14.13 (13.40–15.10)	14.50 (13.70–15.50)	<0.001
**Vitamin D**	67.80 (54.60–85.70)	66.90 (54.30–85.20)	71.10 (56.50–87.40)	0.020
**Lymphocytes** (%)	29.60 (24.60–34.20)	30.00 (25.00–34.50)	28.30 (23.10–32.60)	<0.001
**Eosinophils** (%)	2.50 (1.70–3.50)	2.40 (1.60–3.40)	2.70 (1.70–4.00)	<0.001
**ALT**	23.00 (18.00–29.00)	23.00 (18.00–29.00)	21.00 (17.00–28.00)	0.002
**Smoking status**				<0.001
Never	4092 (51.66)	3666 (57.60)	426 (27.71)	
Former	2336 (29.34)	1760 (27.12)	576 (38.28)	
Current	1633 (19.00)	1115 (15.27)	518 (34.01)	
**Smoking years**	0.00 (0.00–24.00)	0.00 (0.00–18.00)	22.00 (0.00–38.00)	<0.001
**Physical activity**				0.015
Low	4909 (57.36)	4031 (58.36)	878 (53.31)	
Moderate	1759 (23.56)	1411 (23.20)	348 (25.01)	
High	1393 (19.08)	1099 (18.44)	294 (21.67)	
**Asthma**	979 (12.59)	683 (10.75)	296 (20.03)	<0.001
**Diabetes**	1248 (11.24)	1009 (11.16)	239 (11.57)	0.717
**CKD**	630 (5.71)	408 (4.42)	222 (10.89)	<0.001

Data are presented as n (unweighted count) and % (weighted percentage) for categorical variables, and median (interquartile range) for continuous variables. P-values were calculated using the Rao-Scott chi-squared test for categorical variables and the survey-weighted Wilcoxon rank-sum test for continuous variables, accounting for the NHANES complex survey design. COPD, chronic obstructive pulmonary disease. BMI: body mass index. PNI: prognostic nutritional index. ALT: alanine aminotransferase (U/L). CKD: chronic kidney disease. Statistical significance was defined as p<0.05.

### Model evaluation and validation

Performance evaluation of all models was conducted on the independent validation set. This study employed the area under the receiver operating characteristic curve (AUC) as the core metric for model selection, as AUC comprehensively reflects a model’s ability to discriminate between COPD patients and non-COPD individuals across all possible classification thresholds. To comprehensively evaluate and visualize model performance, this study generated multiple graphical representations.

Receiver operating characteristic (ROC) curves were plotted for all 12 models with their respective AUC values annotated, and all curves were consolidated into a single figure to facilitate direct comparison. Forest plots summarized the AUC values and their 95% confidence intervals for all 12 models, enabling straightforward horizontal comparison of model performance differences. Uncertainty around classification metrics was quantified using 1000-iteration bootstrap resampling of the validation and training sets, with the optimal threshold re-derived via Youden’s index within each iteration; 95% confidence intervals were calculated using the percentile method. Residual box plots were generated to assess the distribution characteristics and stability of classification errors across models. Residuals were defined as the actual value (0 or 1) minus the predicted probability, with root mean square error (RMSE) calculated for each model. Calibration was assessed by dividing samples into 10 groups according to predicted probabilities, with scatter plots generated using mean predicted probability as the x-axis and observed COPD incidence rate as the y-axis. The diagonal line represents ideal calibration.

Decision curve analysis (DCA) evaluated the net benefit of models at different risk thresholds, with ‘treat all’ and ‘treat none’ strategies serving as reference baselines. Clinical impact curves were constructed to display the number of cases predicted as high-risk and the actual number of high-risk cases at different thresholds for the optimal model, providing an intuitive assessment of the model’s potential impact in clinical decision-making contexts.

### Model interpretation

To elucidate the underlying mechanisms of the optimal classification model and identify key risk factors, this study performed SHapley Additive exPlanations (SHAP) analysis on the model with the highest AUC. The SHAP method, grounded in Shapley value theory from game theory, quantifies the marginal contribution of each feature to model classifications, thereby providing feature importance assessments that are both globally meaningful and locally interpretable. Multiple visualization plots were generated to interpret model behavior from different perspectives.

The SHAP summary plot (bee swarm plot) displays the distribution of SHAP values for all features, where each point represents a sample, color indicates the magnitude of the feature value, and the x-axis represents the SHAP value (positive values indicate increased COPD risk, while negative values indicate decreased risk). This plot intuitively presents feature importance and the directionality of their relationship with the outcome. The SHAP feature importance plot (bar chart) ranks features according to their mean absolute SHAP values, displayed as horizontal bars with specific numerical annotations, clearly identifying the most important risk factors.

SHAP dependence plots were generated for the top three important features, illustrating the dependence relationship between feature values and SHAP values, which facilitates the identification of nonlinear effects and threshold effects. The SHAP waterfall plot was constructed for representative cases to decompose individual predictions, demonstrating how each feature cumulatively contributes from the baseline value to the final predicted probability, thereby providing a transparent interpretation of prediction results for individual samples.

### Statistical analysis

For descriptive statistics, continuous variables were expressed as median and interquartile range (IQR), while categorical variables were presented as frequencies and percentages. Group comparisons employed appropriate statistical tests, with weighted Mann-Whitney U tests applied to continuous variables and weighted chi-squared tests applied to categorical variables. To ensure that analytical results were representative of the United States population aged ≥40 years, statistical analyses incorporated NHANES-provided sampling weights, with weighted analyses under complex sampling design conducted using the *survey* package in R^9^.

All data processing, statistical analysis, and machine learning modeling in this study were performed using R software (version 4.4.1). Primary R packages utilized included *tidyverse* for data cleaning and processing, *haven* for reading XPT format data files, *survey* for weighted statistical analysis under complex sampling design, *VIM* for missing data visualization, *glmnet* for LASSO regression and variable selection, and *caret* for providing a unified framework and hyperparameter optimization for machine learning models^10,11^. Additional packages including *randomForest, e1071, nnet, gbm, adabag, and pls* were employed to implement various machine learning algorithms. Furthermore, the *xgboost, lightgbm,* and *catboost* packages were used to implement extreme gradient boosting, light gradient boosting machine, and categorical boosting algorithms, respectively. For model evaluation and visualization, the *pROC* package was utilized for ROC curve analysis and AUC calculation, the *rms* package for generating calibration curves, the *dcurves* package for decision curve analysis, the *ggplot2* package for high-quality graphical visualization, and the *gridExtra* and *grid* packages for figure layout and arrangement. For model interpretation, the *kernelshap* and *shapviz* packages were employed for SHAP value calculation and visualization analysis. All statistical tests were two-sided, with p<0.05 considered to indicate statistical significance.

## RESULTS

### Baseline characteristics

Table 1 presents a comparison of baseline characteristics across the overall population, training set, and validation set. Overall, the study participants had a median age of 54 years (IQR: 47–62), with males comprising 48.44% and females 51.56% of the sample. The training and validation sets demonstrated no significant differences in any baseline characteristics (all p>0.05), indicating excellent balance in dataset partitioning and ensuring the reliability of subsequent model evaluation.

Table 2 presents a comparison of baseline characteristics between the COPD and non-COPD groups. Regarding demographic characteristics, COPD patients were significantly older than non-COPD individuals (p<0.001). The proportion of males was significantly higher in the COPD group compared to the non-COPD group (p<0.001). Body mass index (BMI) was significantly lower in the COPD group than in the non-COPD group (p<0.001). Smoking status differed significantly between the two groups (p<0.001). In the COPD group, current smokers comprised 34.01% and former smokers comprised 38.28%, whereas never smokers comprised 57.60% in the non-COPD group. The number of smoking years was significantly higher in the COPD group compared to the non-COPD group, with median values of 22 years and 0 years, respectively (p<0.001).

### LASSO variable selection results

This section presents the variable selection process and final results of LASSO regression. The optimal regularization parameter was determined through repeated 10-fold cross-validation (five repetitions), with lambda.1se selected as the final parameter according to the one-standard-error rule. Supplementary file Figure 1 illustrates the complete LASSO variable selection process. Supplementary file Figure 1a depicts the LASSO coefficient path plot, clearly demonstrating the dynamic trajectory of how regression coefficients for each variable gradually shrink toward zero as the penalty strength (log lambda) increases. Supplementary file Figure 1b shows the cross-validation error curve, displaying the binomial deviance corresponding to different lambda values along with their 95% confidence intervals, with two vertical dashed lines marking the positions of lambda. min and lambda.1se. Based on the lambda.1se criterion, LASSO regression successfully identified 16 variables with non-zero regression coefficients from the 33 candidate feature variables as the final feature set. Table 3 provides a detailed list of these 16 variables and their corresponding LASSO regression coefficients.

**Table 3:** Variables selected by LASSO logistic regression with non-zero coefficients at lambda.1se, NHANES 2007–2012 (training set, N=5643)

*Variables*	*LASSO coefficients*
Age (years)	0.04581
Gender	-0.33522
Race/Ethnicity	0.03950
BMI	-0.04382
PNI	-0.0001
Hemoglobin	0.05001
Vitamin D	0.00129
Lymphocytes	-0.01708
Eosinophils	0.00406
ALT	-0.0009
Smoking status	0.12001
Smoking years	0.02765
Physical activity level	0.01761
Asthma	0.79891
Diabetes	-0.01102
CKD	0.02310

LASSO logistic regression was applied to 33 candidate variables. Sixteen variables with non-zero coefficients were retained. Positive coefficients indicate increased COPD risk; negative coefficients indicate decreased risk. LASSO: Least Absolute Shrinkage and Selection Operator. BMI: body mass index. PNI: prognostic nutritional index. ALT: alanine aminotransferase. CKD: chronic kidney disease.

### Performance comparison of machine learning models

This section systematically compared the classification performance of 12 machine learning models on both training and validation sets. Supplementary file Figure 2 presents the AUC value comparisons and ROC curves for all models. Supplementary file Figures 2a and 2b display forest plots of AUC values for the training and validation sets, respectively, with diamond-shaped points representing the AUC values of each model and horizontal error bars indicating 95% CI, facilitating intuitive comparison of performance differences across models. On the training set, LightGBM demonstrated optimal performance (AUC=0.840), followed by CatBoost (AUC=0.811) and Gradient Boosting Machine (AUC=0.788). On the validation set, CatBoost achieved the highest AUC value (AUC=0.788), outperforming LightGBM (AUC=0.753). Although LightGBM exhibited the best performance on the training set, its inferior performance on the validation set compared to CatBoost suggested potential overfitting. Supplementary file Figures 2c and 2d present the ROC curves for the training and validation sets, respectively. From the morphology of the ROC curves, it is evident that the curves for CatBoost and LightGBM are closest to the upper left corner, indicating the best classification performance.

Supplementary file Figures 3a and 3b present residual box plots for the training and validation sets, respectively. The validation set residual distribution revealed that CatBoost and Logistic Regression models exhibited the most compact residual boxes with smaller RMSE values, suggesting superior classification stability, consistent with their excellent AUC performance.

On the validation set, CatBoost achieved the highest AUC of 0.788 (95% CI: 0.766–0.810). DeLong’s test for pairwise AUC comparison demonstrated that CatBoost significantly outperformed seven models: SVM-RBF (0.727, p<0.001), KNN (0.711, p<0.001), PLS-DA (0.750, p<0.001), XGBoost (0.741, p<0.001), LightGBM (0.753, p<0.001), Random Forest (0.772, p=0.004), and AdaBoost (0.779, p=0.011). No statistically significant differences were observed between CatBoost and Logistic Regression (0.785, p=0.375), Neural Network (0.785, p=0.526), LDA (0.785, p=0.484), or GBM (0.782, p=0.070). Complete pairwise DeLong test results are provided in Supplementary file Table S1.

Table 4 presents performance metrics calculated at model-specific optimal thresholds for both training and validation sets. On the validation set, CATBoost achieved the highest Youden’s index of 0.434 with sensitivity of 0.706 and specificity of 0.728. Validation set sensitivity ranged from 0.607 to 0.763 and specificity from 0.609 to 0.786 across all models.

**Table 4:** Classification performance metrics (value and range) of 12 machine learning models on the training set (N=5643) and validation set (N=2418), evaluated at model-specific optimal thresholds determined by Youden’s index, NHANES 2007–2012

*Model*	*Sensitivity*	*Specificity*	*PPV*	*NPV*	*Accuracy*	*Youden*	*AUC* *95% CI*
**Training set**							
RandomForest	0.745 (0.660–0.781)	0.659 (0.623–0.739)	0.337 (0.318–0.380)	0.918 (0.902–0.928)	0.675 (0.651–0.728)	0.404 (0.380–0.435)	0.768 (0.752–0.784)
GradientBoosting	0.730 (0.670–0.803)	0.698 (0.631–0.757)	0.359 (0.326–0.395)	0.918 (0.906–0.934)	0.704 (0.661–0.742)	0.428 (0.405–0.462)	0.788 (0.773–0.803)
SVM_Kernel	0.612 (0.572–0.667)	0.780 (0.735–0.809)	0.393 (0.356–0.426)	0.896 (0.887–0.907)	0.748 (0.719–0.769)	0.392 (0.365–0.425)	0.726 (0.707–0.745)
LogisticModel	0.680 (0.632–0.809)	0.754 (0.621–0.802)	0.391 (0.327–0.431)	0.910 (0.901–0.935)	0.740 (0.657–0.770)	0.434 (0.409–0.469)	0.787 (0.772–0.802)
NeighborMethod	0.643 (0.615–0.675)	0.684 (0.667–0.697)	0.321 (0.295–0.340)	0.892 (0.882–0.904)	0.676 (0.661–0.688)	0.327 (0.296–0.357)	0.716 (0.699–0.733)
PLSModel	0.752 (0.599–0.797)	0.623 (0.583–0.777)	0.316 (0.299–0.394)	0.915 (0.891–0.927)	0.647 (0.622–0.745)	0.375 (0.351–0.408)	0.757 (0.741–0.773)
BoostingMethod	0.705 (0.647–0.805)	0.724 (0.624–0.780)	0.372 (0.326–0.405)	0.913 (0.904–0.933)	0.720 (0.656–0.754)	0.428 (0.402–0.461)	0.786 (0.771–0.800)
NeuralNet	0.706 (0.617–0.817)	0.705 (0.601–0.792)	0.358 (0.313–0.413)	0.912 (0.898–0.936)	0.705 (0.639–0.762)	0.411 (0.389–0.446)	0.773 (0.758–0.789)
DiscriminantModel	0.739 (0.631–0.814)	0.679 (0.605–0.787)	0.349 (0.317–0.419)	0.918 (0.899–0.935)	0.691 (0.642–0.761)	0.418 (0.395–0.454)	0.785 (0.770–0.800)
XGBoost	0.718 (0.610–0.807)	0.651 (0.567–0.764)	0.323 (0.292–0.380)	0.909 (0.892–0.927)	0.664 (0.607–0.738)	0.369 (0.346–0.403)	0.754 (0.738–0.770)
CATBoost	0.724 (0.690–0.790)	0.748 (0.691–0.774)	0.400 (0.356–0.427)	0.921 (0.913–0.937)	0.743 (0.706–0.763)	0.471 (0.447–0.503)	0.811 (0.797–0.825)
LightGBM	0.725 (0.691–0.797)	0.798 (0.727–0.826)	0.455 (0.398–0.488)	0.926 (0.917–0.939)	0.784 (0.739–0.803)	0.523 (0.495–0.552)	0.840 (0.828–0.853)
**Validation set**							
RandomForest	0.697 (0.573–0.766)	0.712 (0.649–0.837)	0.360 (0.325–0.462)	0.910 (0.889–0.926)	0.709 (0.667–0.790)	0.409 (0.376–0.461)	0.772 (0.748–0.796)
GradientBoosting	0.706 (0.636–0.825)	0.714 (0.597–0.787)	0.365 (0.308–0.418)	0.913 (0.899–0.939)	0.713 (0.637–0.761)	0.420 (0.386–0.471)	0.782 (0.760–0.805)
SVM_Kernel	0.607 (0.522–0.680)	0.786 (0.716–0.861)	0.398 (0.353–0.483)	0.896 (0.880–0.913)	0.753 (0.709–0.801)	0.394 (0.352–0.444)	0.727 (0.699–0.756)
LogisticModel	0.638 (0.605–0.831)	0.786 (0.592–0.812)	0.410 (0.311–0.448)	0.903 (0.893–0.940)	0.758 (0.634–0.779)	0.425 (0.389–0.475)	0.785 (0.762–0.808)
NeighborMethod	0.658 (0.510–0.705)	0.671 (0.647–0.802)	0.317 (0.288–0.385)	0.894 (0.873–0.911)	0.668 (0.647–0.751)	0.329 (0.281–0.377)	0.711 (0.684–0.739)
PLSModel	0.612 (0.490–0.810)	0.747 (0.544–0.865)	0.360 (0.288–0.463)	0.892 (0.876–0.926)	0.721 (0.596–0.796)	0.359 (0.326–0.416)	0.750 (0.725–0.774)
BoostingMethod	0.715 (0.610–0.813)	0.705 (0.618–0.804)	0.361 (0.316–0.422)	0.914 (0.898–0.936)	0.707 (0.649–0.769)	0.420 (0.381–0.468)	0.779 (0.756–0.802)
NeuralNet	0.671 (0.597–0.845)	0.749 (0.564–0.823)	0.383 (0.306–0.455)	0.907 (0.893–0.944)	0.734 (0.616–0.784)	0.420 (0.387–0.469)	0.785 (0.762–0.808)
DiscriminantModel	0.675 (0.626–0.793)	0.756 (0.629–0.806)	0.391 (0.324–0.439)	0.909 (0.897–0.932)	0.741 (0.659–0.773)	0.431 (0.390–0.478)	0.785 (0.762–0.809)
XGBoost	0.763 (0.651–0.810)	0.609 (0.579–0.715)	0.312 (0.287–0.367)	0.917 (0.894–0.933)	0.638 (0.615–0.708)	0.372 (0.336–0.426)	0.741 (0.716–0.765)
CATBoost	0.706 (0.648–0.774)	0.728 (0.667–0.774)	0.377 (0.338–0.423)	0.914 (0.899–0.931)	0.724 (0.684–0.758)	0.434 (0.393–0.486)	0.788 (0.766–0.810)
LightGBM	0.684 (0.524–0.794)	0.687 (0.574–0.856)	0.337 (0.292–0.427)	0.903 (0.885–0.926)	0.687 (0.615–0.787)	0.371 (0.333–0.421)	0.753 (0.728–0.777)

AUC and 95% CI, calculated using DeLong’s method. Pairwise AUC comparisons between CatBoost and all other models using DeLong’s test are presented in Supplementary file Table S1. PPV: positive predictive value. NPV: negative predictive value. Youden: Youden’s index. SVM: support vector machine. PLS: partial least squares. GBM: gradient boosting machine. XGBoost, extreme gradient boosting. CatBoost: categorical boosting. LightGBM: light gradient boosting machine.

### Clinical evaluation of the optimal model

Based on validation set AUC values, CatBoost was identified as the optimal classification model (AUC=0.788), and this section provides an in-depth clinical performance evaluation of this model. Supplementary file Figures 3c and 3d present the calibration curves for the CatBoost model on the training and validation sets, respectively. As shown in the Supplementary file Figures, the CatBoost model demonstrated excellent calibration performance on both datasets, with the vast majority of scatter points falling near the diagonal line, indicating high concordance between the model-predicted COPD risk probabilities and actual incidence rates.

Supplementary file Figures 4a and 4b present the decision curve analysis (DCA) results for all 12 models on the training and validation sets, respectively. From the validation set DCA curve (Supplementary file Figure 4b), it is evident that across a wide range of risk thresholds from 0.1 to 0.6, the decision curves for ensemble learning models such as CatBoost, LightGBM, and Random Forest are positioned above both baseline strategies, indicating that using these models to guide clinical decisions can achieve higher clinical net benefit compared to extreme strategies. The decision curve for the CatBoost model is the smoothest and positioned at the top, suggesting its strongest clinical utility. To facilitate clinical interpretation, we examined the CatBoost model’s performance at specific probability thresholds on the validation set (Supplementary file Table 2). At a 20% threshold, the model identified 823 individuals as high risk, capturing 314 of 456 COPD cases (sensitivity 68.9%) while generating 509 false positives, corresponding to 1.6 false positives per true positive and a positive predictive value of 38.2%. At a more conservative 30% threshold, 485 individuals were flagged, with 225 true positives (sensitivity 49.3%), 260 false positives, and 1.2 false positives per true positive. Compared with a ‘screen all’ strategy, the CatBoost model at the 20% threshold reduced unnecessary referrals for spirometry by approximately 37 per 100 individuals screened while maintaining a positive net benefit.

Supplementary file Figures 4c and 4d present the clinical impact curves for the CatBoost model on the training and validation sets, respectively. These Supplementary file Figures display the number of cases predicted as high-risk by the model (red solid line) and the actual number of high-risk cases (blue dashed line) at different risk thresholds. From the validation set clinical impact curve (Supplementary file Figure 4d), it can be observed that within the risk threshold range of 0.0 to 0.4, the red curve (predicted high-risk number) consistently remains slightly above the blue curve (actual high-risk number), indicating a mild tendency toward overestimation but with overall consistent trends. As the threshold increases above 0.6, both curves gradually approach zero, indicating that the number of cases identified as high-risk decreases sharply at high thresholds, which is consistent with the epidemiological characteristics of relatively low COPD prevalence in the general population.

### Model interpretability analysis

To elucidate the classification mechanisms of the CatBoost model and identify key risk factors, this section employed the SHAP method for model interpretation. Supplementary file Figure 5a presents the SHAP feature importance plot, ranking features according to their mean absolute impact on model predictions The results revealed that age and smoking years were jointly the most important classification features, far exceeding other variables, indicating that these two factors exert the most significant influence on COPD risk. These were followed sequentially by BMI, race/ethnicity, sex, asthma, and smoking status.

Supplementary file Figure 5b displays the SHAP bee swarm plot, illustrating the distribution of SHAP values for all features and their relationship with COPD risk. Key patterns emerged from the analysis. Age and smoking years both demonstrated strong positive correlations with COPD risk, with higher values concentrated in positive SHAP regions. Conversely, high BMI (yellow points) appeared predominantly in negative SHAP regions, suggesting a protective effect of obesity against COPD. Female sex (yellow points) clustered in negative SHAP regions while male sex (purple points) was distributed in positive regions, validating the protective effect of female sex. Both asthma and current smoking status increased COPD risk.

Supplementary file Figure 5c presents SHAP dependence plots for the three most important features (age, smoking years, and BMI). For age, SHAP values increased approximately linearly from negative values at age 40 years to high positive values at age 80 years, with samples having longer smoking duration (yellow points) showing higher SHAP values at the same age, indicating a synergistic effect between age and smoking exposure. Smoking years similarly demonstrated a linear increasing trend, with elderly smokers exhibiting higher SHAP values than younger smokers. The BMI variable revealed a significant U-shaped nonlinear relationship with COPD risk. Low BMI represented a risk factor (positive SHAP values), moderate BMI (30–40 kg/m^2^) conferred protection (lowest SHAP values), while morbid obesity (>40 kg/m^2^) showed diminished protective effects. Color coding by asthma status revealed interaction effects: asthma significantly increased COPD risk at low BMI but appeared to reduce risk in the moderate BMI range.

Supplementary file Figure 5d presents the SHAP waterfall plot for a representative COPD case (Sample 6), demonstrating individual classification decomposition. Smoking years (29 years) contributed most substantially to COPD risk, followed by age (64 years). Female sex reduced the predicted risk (-0.177). After cumulative contribution of all features, the predicted COPD risk for this sample reached approximately 28% [f(x)= -0.917].

## DISCUSSION

In this nationally representative study of 8061 US adults aged ≥40 years, we developed machine learning models for COPD classification that achieved good discrimination (AUC=0.788). Among 12 algorithms evaluated, CatBoost demonstrated optimal performance. Systematic feature selection identified 16 features, with age and smoking duration emerging as dominant risk factors through SHAP analysis. The model revealed complex non-linear relationships, including BMI’s U-shaped association with COPD risk and synergistic age-smoking interactions that conventional regression approaches cannot capture. Decision curve analysis confirmed net clinical benefit across risk thresholds of 0.1–0.6. However, the moderate discrimination and lack of external validation in independent populations limit conclusions about generalizability and readiness for clinical implementation. External validation represents the essential next step before this framework can be considered for clinical application.

The CatBoost model’s AUC of 0.788 represents meaningful improvement over traditional approaches. Systematic reviews of conventional regression models for COPD classification consistently report C-statistics of 0.62–0.77^3,12^, characterized as having moderate but suboptimal discriminative ability for clinical application. This improvement confirms the theoretical advantage of ensemble learning methods in capturing non-linear relationships and complex variable interactions beyond logistic regression’s linear assumptions. However, whether this level of discrimination suffices for clinical implementation requires external validation and end-user assessment. The critical question is not solely whether the model performs better than regression, but whether it performs well enough to change clinical practice.

Several recent machine learning studies report higher AUCs (0.81–0.87) for COPD-related outcomes^13-15^. However, these models typically utilized smaller, selected cohorts from specialized respiratory clinics or tertiary care hospitals where patient populations have higher disease prevalence and more severe phenotypes, potentially introducing selection bias that limits generalizability^4^. The key distinction is that those higher performing models sacrificed population representativeness for classification accuracy in selected samples, while our NHANES-based approach prioritizes broader applicability across the general US population aged ≥40 years. Few existing COPD classification studies have systematically compared more than five algorithms^16^. This comprehensive evaluation of twelve distinct approaches provides evidence-based guidance for algorithm selection, with the consistent superiority of ensemble methods strengthening confidence in these findings. The moderate AUC of 0.788 should be interpreted in context of COPD’s multifactorial etiology involving genetic susceptibility, environmental exposures, and complex gene-environment interactions. No prediction model relying solely on readily available clinical data can fully capture this causal complexity.

Age and smoking duration emerged as the strongest risk factors, consistent with established COPD epidemiology. Age demonstrated an approximately linear relationship with COPD risk across the 40–80 year range, reflecting progressive lung function decline and accumulated oxidative damage. Notably, smoking duration provided stronger discriminative value than simple smoking status categories, aligning with evidence that cumulative exposure time drives pathogenesis more than intensity^17,18^. This suggests duration-based rather than pack-years-based assessment may better capture COPD risk in screening contexts.

Body mass index exhibited a pronounced U-shaped relationship with COPD risk. Low BMI (<20 kg/m^2^) represented a risk factor, moderate overweight (BMI: 25–35 kg/m^2^) conferred protection, while extreme obesity (>40 kg/m^2^) showed diminished protective effects. This pattern reflects the well-documented obesity paradox in COPD^19^. Low BMI indicates cachexia, muscle wasting, and systemic inflammation strongly associated with poor outcomes^20^, while moderate overweight may provide metabolic reserve^21^. However, extreme obesity introduces mechanical constraints from chest wall restriction^22^. This non-linear relationship demonstrates a fundamental limitation of traditional regression approaches that assume monotonic relationships and highlights the value of machine learning methods in capturing complex patterns. Asthma increased COPD risk, reflecting asthma-COPD overlap syndrome characterized by shared inflammatory pathways. Male sex showed higher COPD risk, consistent with historical smoking patterns in birth cohorts represented in NHANES 2007–2012.

The LASSO retention of only the prognostic nutritional index among six candidate inflammatory and nutritional biomarkers warrants interpretation beyond simple exclusion. While NLR, PLR, SII, GNRI, and ALI have demonstrated prognostic value for outcomes in hospitalized COPD patients^23-27^, their exclusion reflects several methodological considerations. First, these composite indices exhibit strong collinearity with constituent parameters already retained by LASSO (hemoglobin, lymphocyte percentage, eosinophil percentage), providing redundant rather than incremental information. Second, biomarkers validated for prognosis (predicting exacerbations and mortality in diagnosed patients) may not necessarily enhance prevalence prediction in general populations. Prognostic and diagnostic models address fundamentally different clinical questions. Third, the conservative lambda.1se strategy intentionally prioritizes parsimony and generalizability over marginal accuracy gains.

Importantly, this finding supports feasibility rather than representing a limitation. The exclusion of specialized biomarkers suggests that pragmatic COPD screening models may not require laboratory tests beyond routine complete blood counts and basic metabolic panels, enhancing implementability in resource-limited primary care settings. Even PNI requires only albumin and lymphocyte count from standard laboratory panels without additional testing costs. Traditional demographic and clinical factors captured the vast majority of discriminative information, suggesting that sophisticated composite biomarkers offer diminishing returns in population-based screening contexts where the goal is efficient case-finding rather than precise prognostication.

SHAP analysis revealed clinically important interaction effects beyond the additive contributions of individual features. Most notably, BMI’s protective effect in the moderate range (25–35 kg/m^2^) was stronger among individuals without asthma, while low BMI (<20 kg/m^2^) combined with asthma substantially elevated COPD risk. This pattern likely reflects asthma-COPD overlap syndrome, where shared inflammatory mechanisms exacerbate airflow limitation in the setting of low body mass^28,29^. Age-smoking duration interactions demonstrated synergistic rather than additive effects, with steeper age-related risk increases among individuals with longer smoking histories. This synergy potentially operates through age-related decline in antioxidant defenses and DNA repair capacity^30,31^. These interaction patterns have direct implications for risk stratification, suggesting that simple additive risk scores may underestimate risk in specific subgroups. Machine learning’s ability to automatically detect such interactions without prior hypothesis specification represents a key advantage over traditional approaches, though careful interpretation remains essential to translate findings into actionable clinical guidance.

Contextualizing our model’s performance against established COPD screening instruments is important for clinical relevance. A systematic review and meta-analysis by Schnieders et al.^23^ reported pooled AUC values of 0.77 (95% CI: 0.63–0.85) for the COPD Population Screener (COPD-PS) questionnaire and 0.72 (95% CI: 0.64–0.78) for the COPD Diagnostic Questionnaire (CDQ). A separate meta-analysis by Gu et al.^33^ similarly reported a pooled AUC of 0.78 for the COPD-PS across eight validation studies. Our CatBoost model (AUC=0.788) demonstrates comparable or slightly superior discrimination relative to these questionnaire-based tools, with the potential advantage of utilizing objective clinical and laboratory measurements rather than self-reported symptoms. However, direct head-to-head comparison using formal reclassification metrics such as the Net Reclassification Index (NRI) and Integrated Discrimination Improvement (IDI) was not feasible because NHANES did not administer these screening questionnaires. Future validation studies using cohorts containing both clinical measurements and established questionnaire responses would enable such a formal comparative evaluation.

Several methodological strengths warrant emphasis. NHANES 2007-2012 data with complex multistage probability sampling ensure findings generalize to the non-institutionalized US population aged ≥40 years, a key advantage over single-center studies. Unlike respiratory specialty clinics enriched for severe disease, NHANES captures the full disease severity spectrum, including undiagnosed cases in community-dwelling populations. Restricting analysis to Grade A and B spirometry according to ATS/ERS standards ensured diagnostic accuracy by excluding technically inadequate measurements that could introduce outcome misclassification. The LASSO approach with repeated cross-validation addressed high-dimensional data challenges, with lambda.1se selection prioritizing parsimony and generalizability over marginal accuracy gains. This conservative strategy guards against overfitting and enhances model transportability to external populations. Comprehensive comparison of twelve algorithms spanning multiple machine learning families provides robust evidence for ensemble method superiority.

Translation to clinical practice requires substantial additional validation work. The model’s moderate discrimination (AUC=0.788) and lack of external validation place it at an early development stage unsuitable for immediate clinical deployment. External validation in diverse, independent populations across different geographical regions and healthcare settings represents the essential prerequisite before clinical implementation can be considered. Prospective validation studies assessing the prediction of incident COPD, rather than prevalent disease, would strengthen causal inference and clinical utility.

Should future validation demonstrate robust and transportable performance, potential applications in primary care settings could be envisioned. Spirometry remains severely underutilized in primary care despite being the diagnostic gold standard, with only 30–50% of newly diagnosed COPD patients undergoing spirometry^34,35^. A validated classification model could be implemented as a web-based risk calculator or integrated into electronic health records to automatically flag high-risk patients during routine encounters. This could help allocate scarce spirometry resources toward individuals most likely to benefit, improving screening efficiency. However, comparative effectiveness research evaluating model-guided screening versus usual care would be necessary to demonstrate actual clinical impact and cost-effectiveness before widespread implementation. All discussion of clinical implementation remains contingent upon successful completion of these validation and effectiveness studies.

### Limitations

This study has several important limitations. Most critically, the lack of external validation in independent cohorts, limits conclusions about generalizability. All performance metrics derive from internal validation using train-test splitting from the same NHANES cycles, which cannot assess performance when applied to different populations, geographical regions, or time periods. External validation in diverse settings is essential before clinical implementation. The cross-sectional design precludes establishing temporal relationships or inferring causality. The model classifies concurrent rather than incident COPD, limiting its utility for primary prevention. Outcome misclassification is possible despite Grade A/B spirometry quality standards, as the GOLD fixed-ratio criterion (FEV_1_/FVC <0.70) may overdiagnose COPD in older adults. Additionally, machine learning models were trained without survey sampling weights, which may limit the representativeness of model parameters for the broader US population, although the impact on individual-level discriminative performance is expected to be modest. Additional limitations include unmeasured confounders (detailed occupational exposures, residential air pollution, biomass fuel exposure, childhood respiratory infections, and genetic variants), which likely contribute to the moderate AUC of 0.788.

## CONCLUSIONS

This study developed a machine learning framework for COPD classification using nationally representative NHANES data (n=8061). CatBoost achieved good discrimination (AUC=0.788) with interpretable predictions through SHAP analysis, identifying age, smoking duration, BMI, and asthma as dominant risk factors, while revealing complex non-linear relationships that conventional regression approaches cannot capture.

However, moderate performance, cross-sectional design, and lack of external validation preclude immediate clinical deployment. External validation in independent populations represents the essential next step. If validated, this framework could facilitate risk-stratified spirometry referral in primary care settings.

## Supplementary Material



## Data Availability

All study data are available for download from the NHANES website (https://wwwn.cdc.gov/nchs/nhanes/Default.aspx).
