# Supplementary material for: An interpretable ensemble learning framework for COPD classification using population-based clinical and laboratory data from NHANES
Source: Tob Induc Dis. 2026 Jul 23;24:10.18332/tid/225230. doi: 10.18332/tid/225230 (PMC13401251; doi:10.18332/tid/225230)
Supplement: Supplementary file 1 [file TID-24-121-s1.pdf]

Tobacco Induced Diseases

Supplementary file

© 2026 Tong H.

DOI:10.18332/tid/225230

The content has been provided by the author(s) and has not been reviewed, verified, or endorsed by European Publishing. It may not have undergone peer review. The views, opinions, and recommendations expressed are solely those of the author(s) and do not necessarily reflect the position of European Publishing. European Publishing accepts no responsibility or liability for any consequences arising from the use of, or reliance on, this content.

Figure 1. LASSO variable selection process for identifying optimal features of COPD (NHANES 2007–2012, training set, n = 5643)

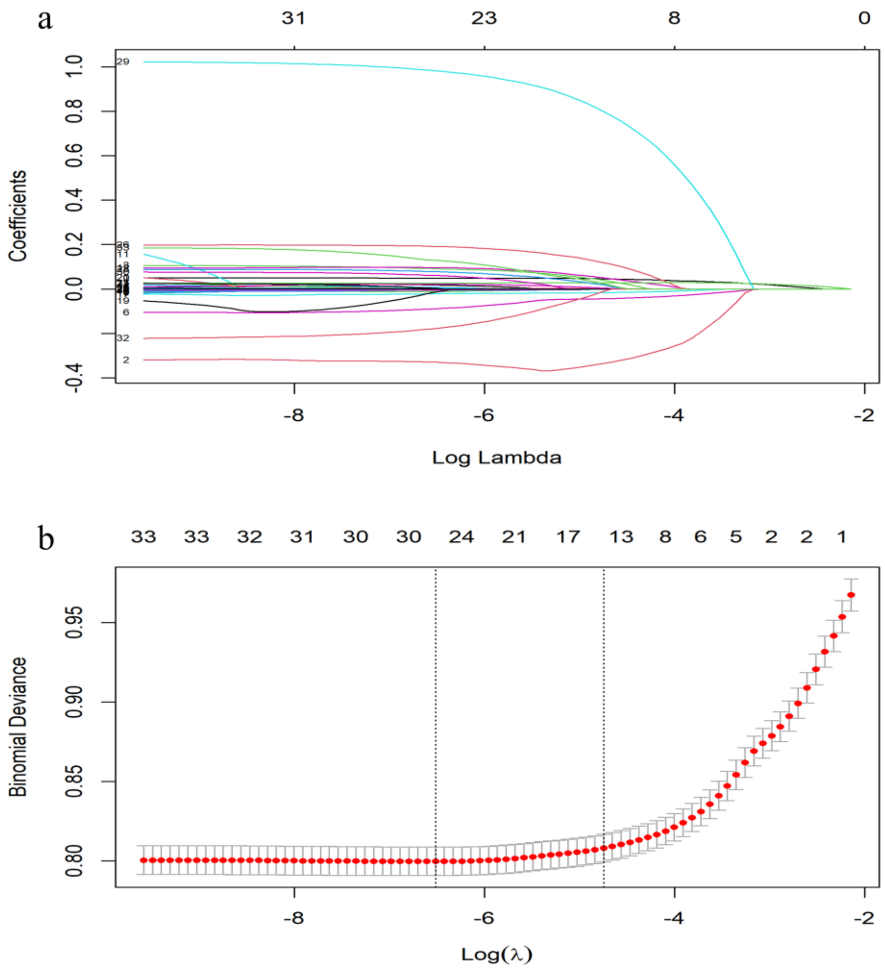

(a) Coefficient path plot showing regression coefficients shrinking toward zero as regularization strength (log λ) increases.

(b) Cross-validation error curve with binomial deviance across log(λ) values; vertical dashed lines indicate lambda.min and lambda.1se. Sixteen variables with non-zero coefficients were retained at lambda.1se.

Figure 2. Discriminative performance of 12 machine learning models on the training set (n = 5643) and validation set (n = 2418)

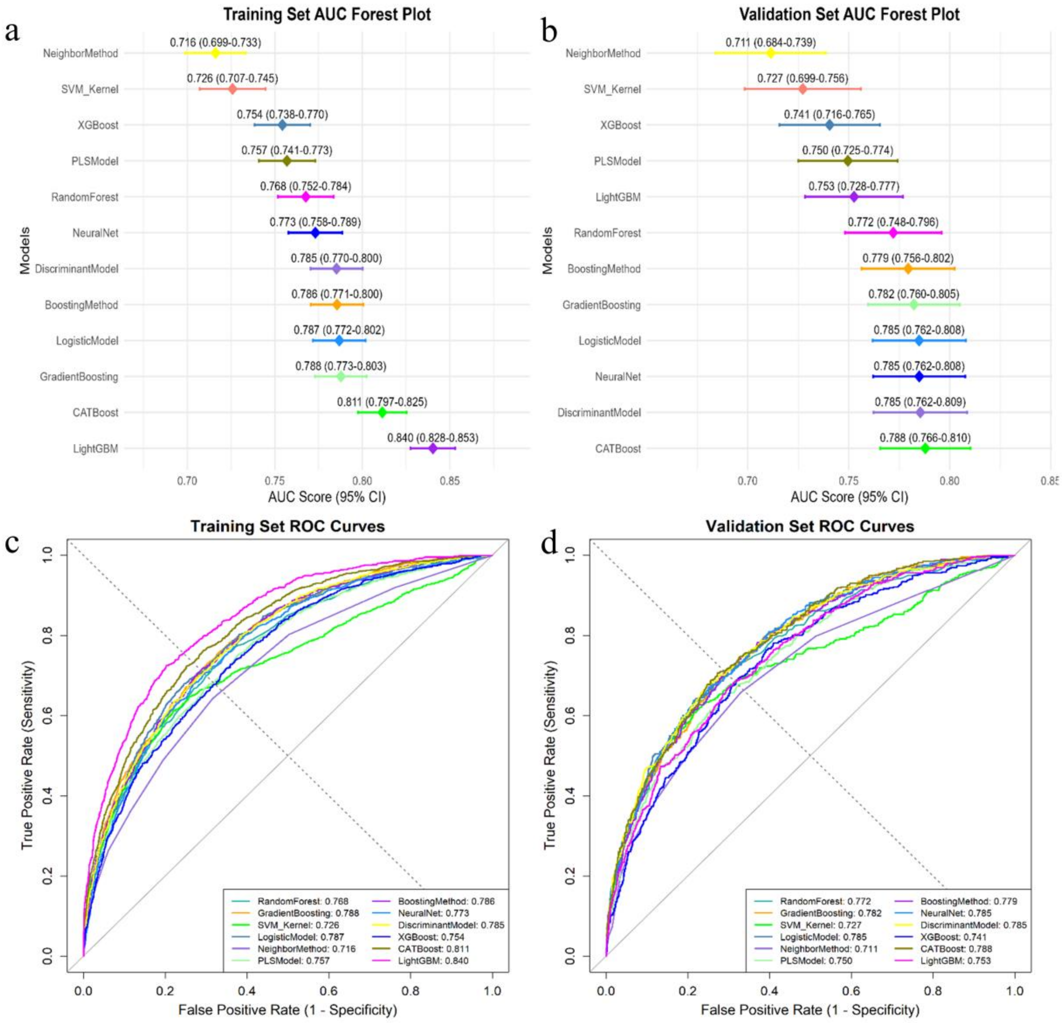

(a) Forest plot of AUC values (95% CI) on the training set. (b) Forest plot of AUC values (95% CI) on the validation set. (c) ROC curves on the training set. (d) ROC curves on the validation set.

AUC, area under the receiver operating characteristic curve; CI, confidence interval; ROC, receiver operating characteristic.

Figure 3. Calibration Performance and Residual Analysis of Machine Learning Models (NHANES 2007–2012)

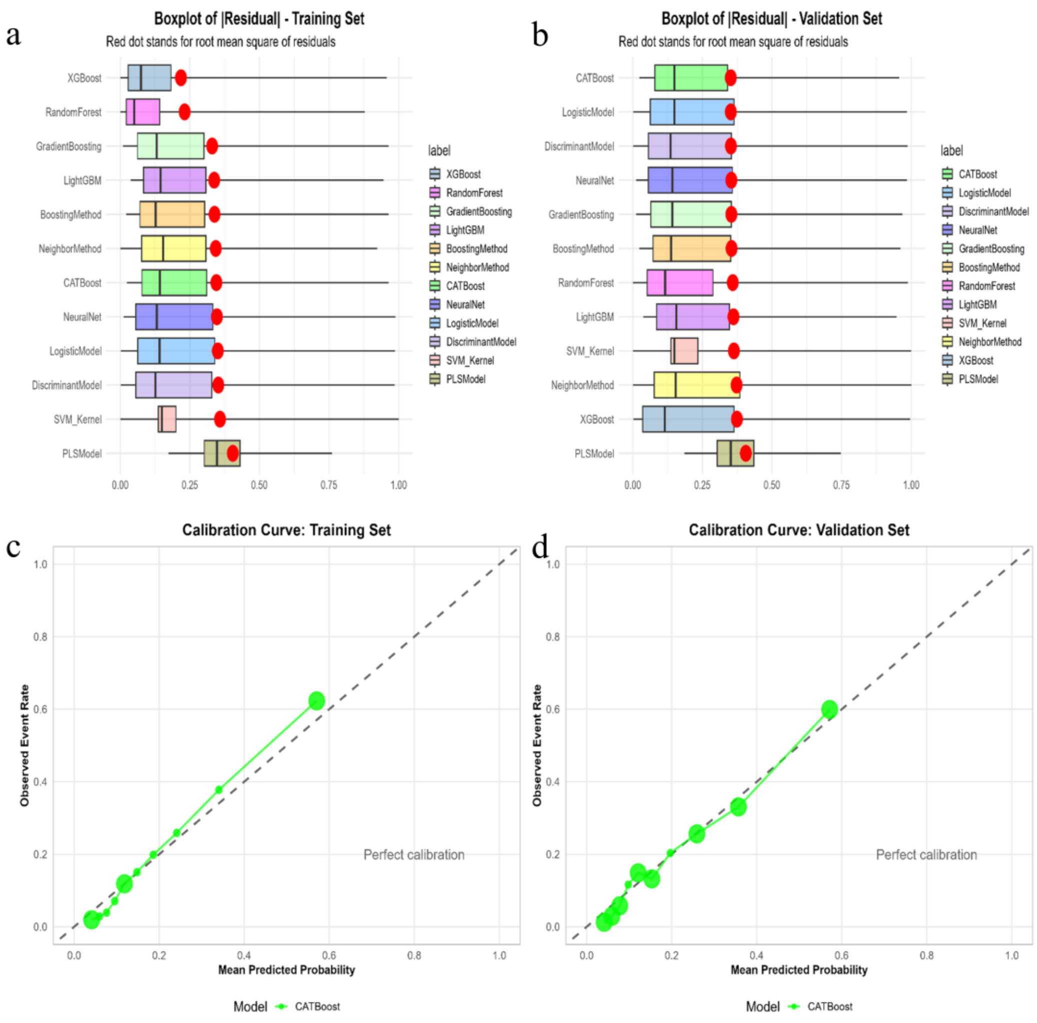

(a) Residual box plots on the training set. (b) Residual box plots on the validation set. Red dots indicate root mean square error (RMSE). Residuals = actual value (0 or 1) minus predicted probability. (c) Calibration curve of the CatBoost model on the training set. (d) Calibration curve on the validation set. Samples were divided into 10 groups by predicted probability; the dashed diagonal represents perfect calibration.

Figure 4. Clinical Utility Assessment of Machine Learning Models (NHANES 2007–2012)

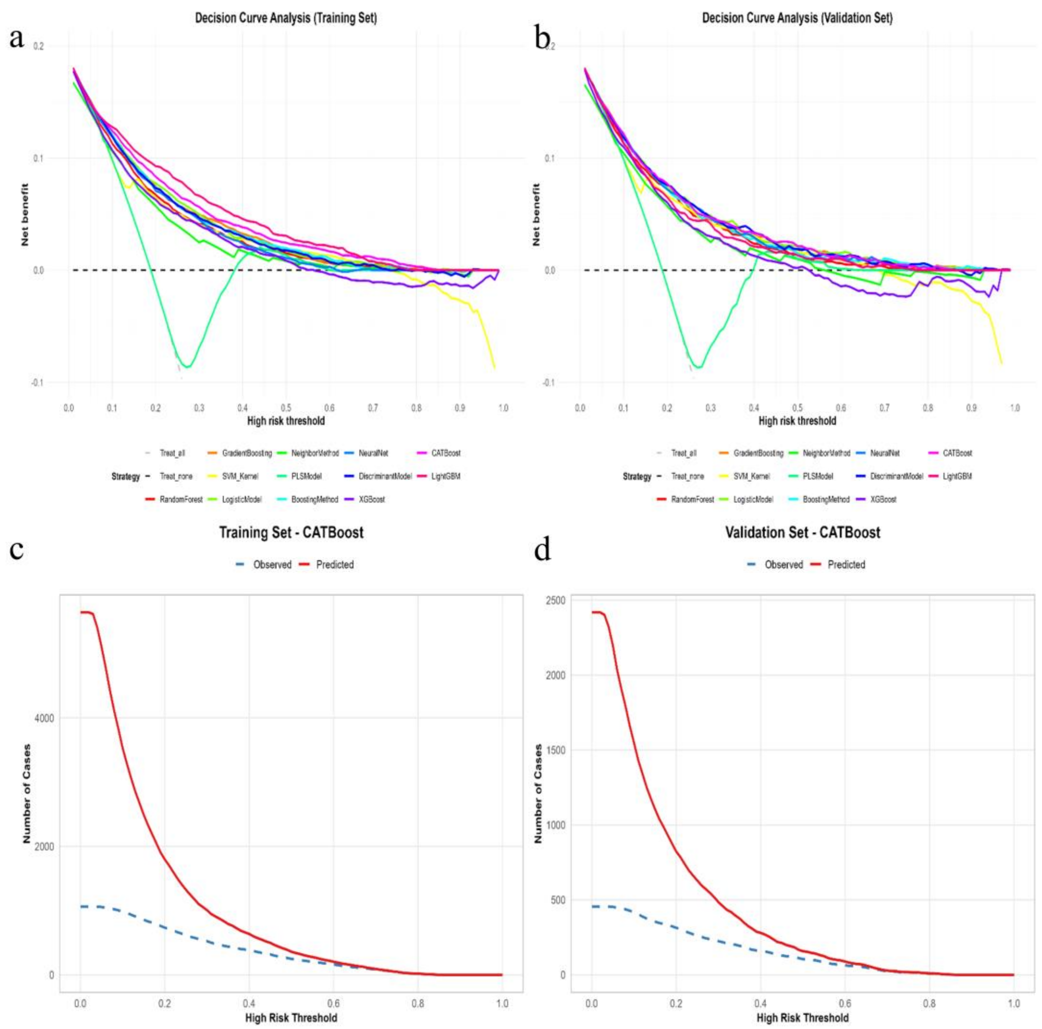

(a) Decision curve analysis (DCA) on the training set. (b) DCA on the validation set. The x-axis represents the high-risk threshold, the y-axis represents net benefit, and the dashed lines represent "treat all" and "treat none" strategies. (c) Clinical impact curve of the CatBoost model on the training set. (d) Clinical impact curve on the validation set; the red solid line indicates the number predicted as high-risk and the blue dashed line indicates actual positives at each threshold.

DCA, decision curve analysis.

Figure 5. SHAP Analysis for Interpretability of the CatBoost Model (NHANES 2007–2012)

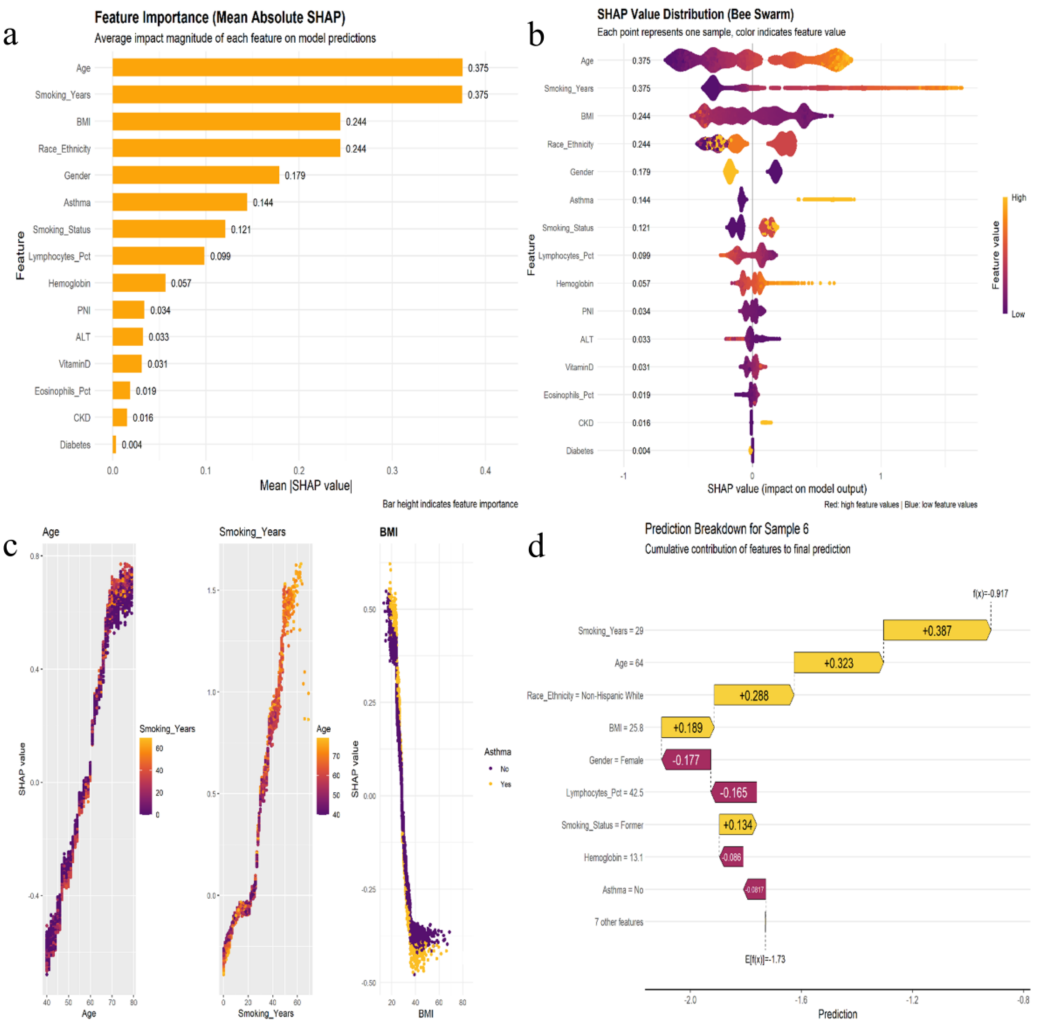

(a) Feature importance ranked by mean absolute SHAP value. (b) Bee swarm plot showing the distribution of SHAP values for each feature; color indicates feature value (yellow = high, purple = low), and the x-axis represents the SHAP value (positive = increased COPD risk). (c) Dependence plots for the top three features (age, smoking years, BMI), with color indicating the strongest interacting feature. (d) Waterfall plot decomposing the prediction for a representative individual sample.

SHAP, SHapley Additive exPlanations; BMI, body mass index.

Table S1. Pairwise DeLong's test for AUC comparison between CatBoost and other machine learning models on the validation set (n = 2418)

Comparison	AUC_CatBoost	AUC_Other	AUC_Difference	Z_statistic	*P*_value	Significant
CatBoost vs RandomForest	0.788	0.772	0.016	2.878	0.004	Yes
CatBoost vs GradientBoosting	0.788	0.782	0.006	1.814	0.07	No
CatBoost vs SVM_Kernel	0.788	0.727	0.061	5.838	<0.001	Yes
CatBoost vs LogisticModel	0.788	0.785	0.003	0.887	0.375	No
CatBoost vs NeighborMethod	0.788	0.711	0.076	6.853	<0.001	Yes
CatBoost vs PLSModel	0.788	0.75	0.038	5.866	<0.001	Yes
CatBoost vs BoostingMethod	0.788	0.779	0.008	2.541	0.011	Yes
CatBoost vs NeuralNet	0.788	0.785	0.003	0.634	0.526	No
CatBoost vs DiscriminantModel	0.788	0.785	0.003	0.7	0.484	No
CatBoost vs XGBoost	0.788	0.741	0.047	6.093	<0.001	Yes
CatBoost vs LightGBM	0.788	0.753	0.035	5.686	<0.001	Yes

AUC comparisons were performed using DeLong's nonparametric test for two correlated ROC curves. *P* values < 0.05 indicate statistically significant differences.

Table S2. Clinical performance of the CatBoost model at selected probability thresholds on the validation set (n = 2418)

Threshold	Flagged_High_Risk	True_Positives	False_Positives	Missed_Cases	Sensitivity	Specificity	PPV	FP_per_TP	NNS	Net_Benefit	NB_Treat_All	Reduction_Unnecessary_per_100
10%	1548	420	1128	36	92.10%	42.50%	27.10%	2.7	3.7	0.1219	0.0984	21.1
15%	1104	355	749	101	77.90%	61.80%	32.20%	2.1	3.1	0.0922	0.0454	26.5
20%	823	314	509	142	68.90%	74.10%	38.20%	1.6	2.6	0.0772	-0.0143	36.6
25%	629	266	363	190	58.30%	81.50%	42.30%	1.4	2.4	0.06	-0.0819	42.6
30%	485	225	260	231	49.30%	86.70%	46.40%	1.2	2.2	0.047	-0.1592	48.1
40%	280	160	120	296	35.10%	93.90%	57.10%	0.8	1.8	0.0331	-0.3524	57.8
50%	158	105	53	351	23.00%	97.30%	66.50%	0.5	1.5	0.0215	-0.6228	64.4
60%	89	64	25	392	14.00%	98.70%	71.90%	0.4	1.4	0.011	-1.0285	69.3

The validation set included 456 COPD cases (18.9%) and 1,962 non-COPD controls. Threshold refers to the predicted probability above which an individual is classified as high risk. FP per TP, number of false positives per true positive; net benefit calculated as TP/N − FP/N × [threshold/(1 − threshold)]. Compared with a "screen all" strategy at the 20% threshold, the model reduced unnecessary spirometry referrals by approximately 37 per 100 individuals screened.
